# Vitamin D and Incidence of Prediabetes or Type 2 Diabetes: A Four-Year Follow-Up Community-Based Study

**DOI:** 10.1155/2018/1926308

**Published:** 2018-03-18

**Authors:** Yun Gao, Tianpeng Zheng, Xingwu Ran, Yan Ren, Tao Chen, Li Zhong, Donge Yan, Fangfang Yan, Qianlin Wu, Haoming Tian

**Affiliations:** ^1^Department of Endocrinology and Metabolism, West China Hospital of Sichuan University, Chengdu 610041, China; ^2^Department of Endocrinology and Metabolism, Affiliated Hospital of Guilin Medical University, Guangxi 541001, China; ^3^Department of Endocrinology and Metabolism, First People's Hospital of Long Quanyi District, Chengdu 610100, China; ^4^Cardiovascular Medicine Department, Chengdu First People's Hospital, Chengdu 610016, China

## Abstract

**Aim:**

To examine whether the baseline 25-hydroxyvitamin D [25(OH)D] level was predictive of the onset of prediabetes or type 2 diabetes (T2DM) in the Chinese population.

**Methods:**

This was a 4-year follow-up study that was conducted in the Chengdu region of China as part of the China National Diabetes and Metabolic Disorders Study. The study included 490 participants that were free of prediabetes and type 2 diabetes mellitus (T2DM) at baseline and had complete data by follow-up examinations. Glucose, insulin, and 25(OH)D levels were measured at baseline and at 4 years later. Prediabetes and T2DM were defined by results obtained from an oral glucose tolerance test.

**Results:**

Over a 4-year follow-up, 95 (48.5‰) developed prediabetes and 31 (15.8‰) individuals developed diabetes. Low 25(OH)D status was significantly associated with the risk of developing prediabetes [OR 3.01 (95% CI: 1.50–6.06), *P* = 0.002] and T2DM [OR 5.61 (95% CI: 1.73–18.27), *P* = 0.004] after adjustment for multiple potential confounders. In a multiple linear regression analysis, low baseline levels of 25(OH)D were an independent predictor of increased insulin resistance over a 4-year period (*P* < 0.05).

**Conclusions:**

The current prospective study suggests that low 25(OH)D levels might have contributed to the incidence of prediabetes or T2DM in Chinese individuals. This trial is registered with TR-CCH-ChiCTR-OCS-09000361.

## 1. Introduction

Type 2 diabetes mellitus (T2DM) is a heterogeneous group of disorders resulting from the combination of genetic predisposition, behavioral, nutritional, and environmental risk factors [1]. The pathogenesis of T2DM involves the development of a relative deficiency in insulin secretion and insulin resistance [1]. There is strong evidence that nutritional risk factors play an important role in pancreatic *β*-cell physiology with emphasis on their effects on insulin secretion [2]. The modifiable risk factors like the nutrients related to *β*-cell dysfunction may be manipulated as an effective way to treat and/or prevent diabetes mellitus.

Vitamin D, as a critical and essential micronutrient for human health, has received widespread attention for numerous nonskeletal effects, including its potential in pancreatic insulin secretion and insulin action [3]. Epidemiological studies indicate that vitamin D deficiency is widespread in those with diabetes [4]. There is also ample evidence to suggest that a low level of serum 25-hydroxyvitamin D [25(OH)D], a generally accepted indicator of vitamin D status, is inversely associated with impaired glucose tolerance (IGT) and diabetes [5–7]. Moreover, higher vitamin D intakes are significantly associated with a lower risk of type 2 diabetes (T2DM) [8] and vitamin D and calcium supplementation can even improve glucose homeostasis in adults with impaired fasting glucose (IFG) [9].

In China, Chengdu plain has one of the lowest annual sunshine totals nationally and most days are cloudy and overcast even if without rain. Hypovitaminosis D is common in the area of the population inhabiting this region. However, little is known regarding whether vitamin D insufficiency or deficiency plays an important role in the heightened prevalence of prediabetes and diabetes among individuals in China. Further, several clinical trials showed that 25(OH)D levels might have no effect on glycemia or the incidence of diabetes [10, 11].

Therefore, the aim of our study was to evaluate the relationship between circulating vitamin D and incident prediabetes or T2DM in the Chinese population living in Chengdu plain.

## 2. Materials and Methods

### 2.1. Study Population

The participants in this study were aged 20–74 years and were enrolled in the China National Diabetes and Metabolic Disorders Study [12], which was a 4-year follow-up study that aimed to determine the prevalence and incidence of T2DM and metabolic disorders. Subjects registered were permanent residents in Yulin Community and Long Quanyi District within Chengdu of Sichuan province from June to August 2007.

Among the 856 participants considered, 174 were excluded because they already presented with hyperglycemia at baseline; 132 were lost to follow-up and 6 subjects died; 42 were excluded because of liver dysfunction, renal dysfunction, cancer, severe gastrointestinal disorders, or were taking vitamin D supplements; 12 were excluded because of incomplete data. This left 490 participants to be included in the present study.

The study was approved by the Drugs/Medical Apparatus and Instruments Ethics Committee at the China Japan Friendship Hospital (07020470055); all procedures performed in studies involving human participants were in accordance with the ethical standards of the institutional and/or national research committee and with the 1964 Helsinki declaration and its later amendments or comparable ethical standards, and all subjects gave their informed consent.

### 2.2. Assessment of Vitamin D Status

Fasting blood samples were collected from June to August 2007, and serum samples were stored at −80°C until assayed. The season selected for blood collection was always in the summer. Vitamin D status was measured as serum 25(OH)D using enzyme immunoassay kits (IDS Ltd., UK). The intra-assay and interassay coefficients of variation for 25(OH)D was 4.7% and 5.5%, respectively. Serum 25(OH)D was measured in the 682 participants without prediabetes or diabetes at baseline. After four years of follow-up, 490 subjects who met the inclusion criteria were finally analyzed in this study.

### 2.3. Ascertainment of Incident Diabetes

The incidence of diabetes at follow-up was defined by undergoing an oral glucose-tolerance test [fasting plasma glucose (FPG) ≥7 mmol/L or a 2-hour plasma glucose (2 h–PG) test post-OGTT ≥ 11.1 mmol/L], or treatment with insulin or oral hypoglycemic agents.

### 2.4. Questionnaire and Anthropometrics

Trained staff administered a standardized questionnaire to each subject. Demographic characteristics, life style risk factors, family history, medical history, and anthropometric parameters were collected during the survey. Further details of these data have been described in our previous study [13].

### 2.5. Diagnostic Criteria

The 1999 World Health Organization (WHO) diagnostic criteria were used to diagnose diabetes, IFG, and IGT [14]. Prediabetes was defined as either IFG or IGT. Hyperglycemia included a diagnosis of diabetes or prediabetes. The homeostasis model assessment of insulin resistance (HOMA-IR) and insulin sensitive index composite (ISIcomp) were used to estimate insulin sensitivity. The *β*-cell function was quantified as the ratio of the incremental insulin to glucose responses over the first 30 min during the OGTT (ΔI30/ΔG30). The latter was also adjusted for insulin sensitivity as it modulates *β*-cell function [(ΔI30/ΔG30)/HOMA-IR] [15].

### 2.6. Statistical Analysis

Baseline characteristics are presented according to quartiles of baseline 25(OH)D levels. Categorical data are presented as percentages and continuous variables as means ± one standard deviation about the mean (normally distributed variables) or as medians with interquartile ranges (skewed variables). Variables following a nonnormal distribution were logarithmically transformed (natural logarithm) before use in parametric analyses. The Pearson's chi-square test was used to test differences for baseline categorical variables. The differences in continuous variables between 25(OH)D groups were tested by analysis of variance (ANOVA). Logistic regression analyses were performed with incident prediabetes or T2DM as the binary outcome variable and 25(OH)D groups as the explanatory variable. The association between the 25(OH)D level at baseline and the change in insulin resistance or *β*-cell function was quantified by multiple linear regression. The SPSS version 16.0 (SPSS Inc., Chicago, IL) statistical software program was used to perform statistical analyses. An alpha value of *P* < 0.05 was considered to indicate statistical significance.

## 3. Results

**Figure 1:**
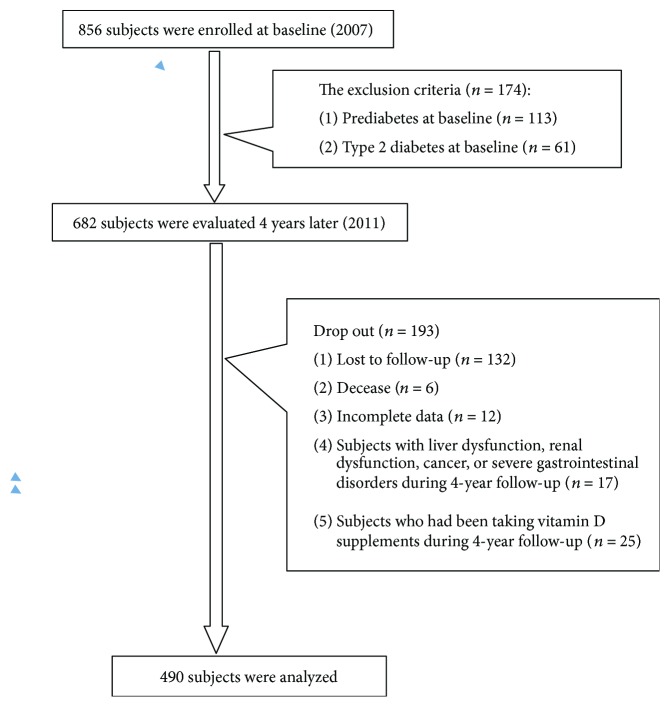
Flow chart of study participants in the study cohort.

Figure 1 shows the flow of participants across the study. At baseline, 856 subjects aged 18–70 years were included, of which, 718 had a second examination four years later. Of these, 490 subjects (180 males and 310 females) who were free of known diabetes or prediabetes at baseline were divided into three groups on the basis of their glucose metabolism status at the end of a four-year follow-up [i.e., normal glucose tolerance (NGT), prediabetes, or T2DM].

The current study participants were aged 45.8 ± 13.5 years, and 63.3% of the subjects recruited were women. The range of 25(OH)D levels was 13.93–80.30 nmol/L. In Table 1, the clinical characteristics and biochemical variables were compared according to quartiles of baseline 25(OH)D levels. At baseline, no significant differences in plasma glucose and insulin concentration obtained during the OGTT were found between the 4 groups. Moreover, insulin sensitivity (including HOMA-IR and ISIcomp) and *β*-cell function (including ΔI30/ΔG30 and (ΔI30/ΔG30)/HOMA-IR) were also comparable between the 4 groups.

There were 1937 person-years of follow-up with an average follow-up period of 3.95 years. During the follow-up period, 95 (19.4%) subjects developed prediabetes (including isolated IFG, isolated IGT, or IFG plus IGT), 31 (6.3%) subjects developed T2DM, and 364 subjects remained normoglycemic. The incidence of prediabetes and T2DM was 48.5 events per 1000 patient-years and 15.8 events per 1000 patient-years, respectively. The 4-year longitudinal study showed that low 25(OH)D levels were associated with the risk of onset of prediabetes [OR 3.01(95% CI 1.50–6.06), *P* = 0.002] and T2DM [OR 5.61 (95% CI 1.73–18.27), *P* = 0.004] in the analyses that were adjusted for age, gender, family history of diabetes, and changes in the values for BMI, WHR, SBP, DBP, TG, and TC and in physical activity, smoking status, and alcohol consumption during follow-up (Table 2).

Low baseline levels of 25(OH)D were an independent predictor of an increase in HOMA-IR and a decrease in the ISIcomp in subjects who developed hyperglycemia, including prediabetes and T2DM, after adjusting for confounding factors (*P* < 0.05; Table 3).

## 4. Discussion

In this study, we prospectively reported that low 25(OH)D levels predicted the onset of prediabetes and T2DM in the Chinese population. These data suggest that 25(OH)D may be involved in the development and pathophysiology of prediabetes and T2DM. Additionally, apart from the associations between baseline 25(OH)D levels and incidences of prediabetes and diabetes, we also assessed the relationship between baseline 25(OH)D levels and changes in insulin resistance and *β*-cell function at follow-up.

Previous studies have demonstrated that the level of 25(OH)D was significantly lower in human subjects with impaired glucose tolerance or T2DM [16, 17]. In this study, we also found that subjects who developed prediabetes or T2DM 4 years later were prone to lower 25(OH)D levels at baseline (data not shown). Therefore, we extrapolate that the reduction in circulating 25(OH)D levels in a Chinese population occurred before the onset of prediabetes or T2DM.

In the 4-year prospective analysis, logistic regression models further revealed that low 25(OH)D levels were independently predictive of the development of hyperglycemia, including prediabetes and T2DM. At the end of follow-up, the normoglycemic subjects with the lower quartile of 25(OH)D at baseline had a higher risk of prediabetes and T2DM, as compared with those with the upper quartile of 25(OH)D levels. The adverse effects of low serum vitamin D levels on increasing prediabetes or T2DM risks may be related to its effect on promoting *β*-cell function and insulin sensitivity [18, 19]. We found that low 25(OH)D levels were an independent predictor of an increase in insulin resistance, as assessed by HOMA-IR and ISIcomp, in subjects who developed hyperglycemia, including prediabetes and T2DM. Similarly, a significant inverse association has been found between the baseline 25(OH)D level and the risk of hyperglycemia and insulin resistance in the Ely population-based prospective study [20]. Moreover, the Australian Diabetes, Obesity and Lifestyle study has also proposed that higher serum 25OHD levels were associated with improved insulin sensitivity, as assessed by HOMA-S [21]. From a physiologic perspective point of view, 1,25-dihydroxyvitamin D can stimulate the pancreatic *β*-cell to secrete insulin [22]. Vitamin D can also reduce inflammation, maintain the levels of Ca^2+^ and ROS at their normal low physiological levels, and maintain the epigenome, through which it can alleviate the onset of insulin resistance [23]. Additionally, vitamin D can stimulate the expression of the insulin receptor and thereby enhance insulin responsiveness for glucose transport [24]. So, vitamin D deficiency may result in a decreased insulin release and insulin resistance and then contributes to the onset of diabetes. Data from observational studies, especially several longitudinal observational cohort studies [25, 26], showed the inverse association between 25(OH)D concentration and incident diabetes and indicated that 25(OH)D concentration was a strong biomarker of diabetes risk. Similar result has been observed in our cohort study.

In the past, it has been suggested that 25(OH)D levels varied with age, ethnicity, genetic background, dietary intake of vitamin D, and so on. Hypovitaminosis D is a common condition in older people [27]. Moreover, some ethnic groups have lower vitamin D levels, which may be due to their skin pigmentation [28]. Individuals with darker skin pigmentation are more likely to have low 25(OH)D [4]. Other genetic factors, such as genetic polymorphisms of vitamin D-binding protein (DBP) or vitamin D receptor (VDR), may affect vitamin D status and may play a role in impaired glucose tolerance or T2DM. DBP polymorphisms can influence bioactive 25(OH)D levels through changes in the ratio of free/bound hormones [29]. Moreover, DBP polymorphisms 1S and 2 were associated with higher fasting plasma insulin in Japanese [30]. Research by Ortlepp et al. indicated that the BB genotype of Bsml VDR polymorphisms in men was associated with higher fasting glucose [31]. In addition, low 25(OH)D levels were linked to increased risk for developing peripheral neuropathy [32], nephropathy [33], and retinopathy [34] in T2DM patients. Therefore, early screening and correction of vitamin D deficiency in the high-risk population might have a beneficial effect on the onset and progress of T2DM.

In this study, we divided the participants into four groups according to quartiles of serum 25(OH)D concentration, instead of the diagnostic criteria of vitamin D deficiency and insufficiency in the Endocrine Society Clinical Practice Guideline, with the overarching objective of studying associations between serum 25(OH)D and the incidence of prediabetes and diabetes. This is because vitamin D deficiency or insufficiency universally exists in the general population that inhabits the Chengdu plain. Only three subjects (0.6%) had serum 25(OH)D ≥ 75 nmol, and seventy-one (14.2%) had serum 25(OH)D ≥ 50 nmol in our study (data not shown).

Some limitations of our study should be considered. First, the gold standard techniques for assessing insulin resistance and *β*-cell function (i.e., a clamp study) were not used in the present study; however, we have used some more common and suitable methods in epidemiological studies, such as HOMA-IR, ISIcomp, ΔI30/ΔG30, and (ΔI30/ΔG30)/HOMA-IR. Second, although all blood samples were taken in the summer season, the data on sun exposure time, use of sunscreen, and dietary habits were not collected in our study. Last, because this was an observational study, residual confounding cannot be excluded and may impact the association of serum 25(OH)D levels with the observed outcomes.

In conclusion, in this prospective study, our data illustrated an independent association between 25(OH)D levels and the onset of hyperglycemia, including prediabetes and T2DM. In addition, 25(OH)D deficiency might increase insulin resistance in apparently healthy Chinese individuals. However, since this is an observational study, further well-designed and large prospective studies and RCT are needed to clarify the role of vitamin D in glucose metabolism and the etiology of T2DM.

## Figures and Tables

**Table 1:** Characteristics at entry according to quartiles of baseline 25(OH)D level in subjects who were initially normoglycemic.

	Q1	Q2	Q3	Q4	P
Range of 25(OH)D (nmol/L)	13.93–33.56	33.57–40.11	40.12–46.38	46.39–80.30	—
*n*	122	123	123	122	—
Percent women *n* (%)	88 (72.1)	79 (64.2)	80 (65.0)	63 (51.6)	0.010
Age (years)	44.8 ± 12.8	45.2 ± 13.8	45.6 ± 13.5	47.8 ± 13.8	0.311
Current smoking *n* (%)	20 (16.4)	28 (22.8)	20 (16.3)	25 (20.5)	0.485
Drinking *n* (%)	28 (23.0)	38 (30.9)	22 (17.9)	36 (29.5)	0.068
Leisure-time physical activity *n* (%)	98 (80.3)	115 (93.5)	98 (79.7)	96 (78.7)	0.005
Family history of diabetes *n* (%)	21 (17.2)	16 (13.0)	12 (9.8)	17 (13.9)	0.397
BMI (kg/m^2^)	23.1 ± 3.2	22.8 ± 3.0	23.5 ± 3.2	23.2 ± 3.1	0.380
WHR	0.85 ± 0.11	0.86 ± 0.08	0.86 ± 0.08	0.86 ± 0.08	0.711
SBP (mmHg)	120.4 ± 18.4	118.5 ± 19.99	115.1 ± 14.6	118.2 ± 17.9	0.145
DBP (mmHg)	76.8 ± 10.2	75.9 ± 10.4	74.1 ± 8.7	76.2 ± 10.1	0.165
FPG (mmol/L)	4.55 ± 0.56	4.62 ± 0.53	4.63 ± 0.49	4.63 ± 0.68	0.268
0.5 h PG (mmol/L)	7.51 ± 1.44	7.74 ± 1.76	7.49 ± 1.66	7.89 ± 1.69	0.158
2 h PG (mmol/L)	5.38 ± 1.16	5.52 ± 1.11	5.45 ± 1.13	5.44 ± 1.02	0.843
TG (mmol/L)	1.23 (0.91, 1.68)	1.12 (0.89, 1.58)	1.17 (0.90, 1.67)	1.12 (0.89, 1.76)	0.842
TC (mmol/L)	5.14 ± 0.96	4.95 ± 0.99	4.95 ± 0.88	4.92 ± 1.03	0.255
LDL-C (mmol/L)	3.04 ± 0.84	2.89 ± 0.79	2.95 ± 0.81	3.00 ± 0.92	0.515
HDL-C (mmol/L)	1.50 ± 0.35	1.47 ± 0.36	1.41 ± 0.32	1.36 ± 0.34	0.005
Fasting insulin (*μ*U/mL)	7.04 (5.67, 8.65)	6.79 (5.37, 8.79)	6.65 (5.31, 8.60)	6.71 (5.44, 8.68)	0.425
0.5 h insulin (*μ*U/mL)	34.22 (19.45, 50.41)	29.74 (19.31, 44.19)	30.66 (18.40, 53.34)	34.70 (20.53, 57.95)	0.720
2 h insulin (*μ*U/mL)	25.89 (17.05, 40.32)	27.26 (18.45, 41.98)	23.88 (16.34, 42.92)	24.02 (16.27, 38.52)	0.672
HOMA-IR	1.63 ± 1.06	1.53 ± 0.69	1.48 ± 0.55	1.51 ± 0.61	0.471
ISIcomp	8.90 ± 3.46	9.39 ± 4.30	9.20 ± 3.31	9.06 ± 3.64	0.862
ΔI30/ΔG30	9.17 (4.67, 14.68)	7.38 (4.36, 16.83)	8.81 (4.10, 16.43)	9.54 (4.34, 17.90)	0.889
(ΔI30/ΔG30)/HOMA-IR	6.04 (3.20, 11.14)	5.68 (3.02, 12.32)	6.49 (3.0, 13.90)	6.88 (3.37, 11.76)	0.835

Data is expressed as mean ± standard deviation, median (interquartile range), or percentage. 25(OH)D: 25-hydroxyvitamin D; Q: quartile; BMI: body mass index; WHR: waist/hip ratio; SBP: systolic blood pressure; DBP: diastolic blood pressure; FPG: fasting plasma glucose; TG: triglyceride; TC: total cholesterol; HDL-C: high-density lipoprotein cholesterol; LDL-C: low-density lipoprotein cholesterol; HOMA-IR: homeostasis model of assessment for insulin resistance index; ISIcomp: insulin sensitive index composite; ΔI30/ΔG30: the ratio of the incremental insulin to glucose responses over the first 30 min during the OGTT.

**Table 2:** Multiple logistic regression analyses of the prospective association between baseline 25(OH)D status and 4-year diabetes incidence.

		Risk of incident prediabetes			Risk of incident diabetes		
		Prediabetes incidence % (*n*/*n*_total_)	OR (95% CI)	*P* value	Diabetes incidence % (*n*/*n*_total_)	OR (95% CI)	*P* value
25(OH)D^†^ (nmol/L)	Q1 (13.93–33.56)	26.2 (32/122)	3.01 (1.50–6.06)	0.002	9.8 (12/122)	5.61 (1.73–18.27)	0.004
	Q2 (33.57–40.11)	18.7 (23/123)	1.68 (0.81–3.46)	0.163	6.5 (8/123)	2.12 (0.64–7.02)	0.217
	Q3 (40.12–46.38)	18.7 (23/123)	1.64 (0.80–3.38)	0.178	4.1 (5/123)	1.77 (0.48–6.58)	0.561
	Q4 (46.39–80.30)	13.9 (17/122)	1.00 (reference)		4.9 (6/122)	1.00 (reference)	
Age	—	—	1.04 (1.02–1.06)	<0.001	—	1.07 (1.03–1.10)	<0.001
FH of diabetes	—	—	3.91 (2.10–7.27)	<0.001	—	4.16 (1.52–11.36)	0.005
Sex (male)	—	—	—	—	—	7.14 (2.86–17.81)	<0.001

^†^OR was calculated with the use of binary logit model (forward conditional). Adjusted for age, sex, FH of diabetes, change in BMI, change in WHR, change in SBP, change in TG, changes in TC, physical activity, smoking status, and alcohol consumption during follow-up. 25(OH)D: 25-hydroxyvitamin D; Q: quartile; FH: family history; WHR: waist/hip ratio; BMI: body mass index; SBP: systolic blood pressure; DBP: diastolic blood pressure; TC: total cholesterol; TG: triglyceride.

**Table 3:** Standardized *β*-coefficients from multiple linear regression analysis of glucose metabolism, insulin resistance, and insulin secretory capacity in the 4-year longitudinal study in subgroups.

		Change in HOMA-IR		Change in ISIcomp		Change in ΔI30/ΔG30		Change in (ΔI30/ΔG30)/HOMA-IR	
		*β*	*P*	*β*	*P*	*β*	*P*	*β*	*P*
Prediabetes^†^	25(OH)D	−0.151	0.001	0.050	0.303	−0.048	0.342	−0.047	0.328
	FH of diabetes	0.142	0.003	−0.127	0.009	−0.089	0.072	−0.099	0.042
	Change in BMI	0.140	0.003	−0.074	0.128	0.048	0.342	−0.009	0.854
	Change in SBP	0.070	0.136	−0.023	0.632	−0.084	0.095	−0.103	0.034
	Change in TG	0.056	0.235	0.013	0.785	0.006	0.913	0.005	0.915
	Change in TC	0.039	0.826	−0.116	0.018	−0.040	0.428	−0.033	0.502
Type 2 diabetes^†^	25(OH)D	−0.203	0.001	0.129	0.006	−0.053	0.277	−0.048	0.306
	FH of diabetes	0.147	0.001	−0.156	0.001	−0.084	0.080	−0.092	0.050
	Change in BMI	0.082	0.066	−0.029	0.538	0.042	0.392	−0.012	0.804
	Change in SBP	0.081	0.073	−0.045	0.331	−0.082	0.089	−0.099	0.035
	Change in TG	0.089	0.050	0.006	0.906	−0.042	0.386	0.007	0.875
	Change in TC	0.069	0.125	−0.129	0.006	0.009	0.179	−0.035	0.455

*β*-coefficient was calculated with the use of multiple linear regression analysis (forward). ^†^Independent variables included in the regression model: baseline 25(OH)D level, age, sex, FH of diabetes, change in BMI, change in WHR, change in SBP, change in TG, and change in TC, physical activity, smoking status, and alcohol consumption during follow-up. 25(OH)D: 25-hydroxyvitamin D; FH: family history; WHR: waist/hip ratio; BMI: body mass index; SBP: systolic blood pressure; TC: total cholesterol; TG: triglyceride; HOMA-IR: homeostasis model of assessment for insulin resistance index; ISIcomp: insulin sensitive index composite; ΔI30/ΔG30: the ratio of the incremental insulin to glucose responses over the first 30 min during the OGTT.

## References

[1] Hu F. B. (2011). Globalization of diabetes: the role of diet, lifestyle, and genes. *Diabetes Care*.

[2] Moore W. T., Bowser S. M., Fausnacht D. W., Staley L. L., Suh K. S., Liu D. (2015). Beta cell function and the nutritional state: dietary factors that influence insulin secretion. *Current Diabetes Reports*.

[3] Rosen C. J., Adams J. S., Bikle D. D. (2012). The nonskeletal effects of vitamin D: an Endocrine Society scientific statement. *Endocrine Reviews*.

[4] Holick M. F. (2007). Vitamin D deficiency. *The New England Journal of Medicine*.

[5] Pittas A. G., Chung M., Trikalinos T. (2010). Systematic review: vitamin D and cardiometabolic outcomes. *Annals of Internal Medicine*.

[6] Forouhi N. G., Ye Z., Rickard A. P. (2012). Circulating 25-hydroxyvitamin D concentration and the risk of type 2 diabetes: results from the European prospective investigation into cancer (EPIC)-Norfolk cohort and updated meta-analysis of prospective studies. *Diabetologia*.

[7] Deleskog A., Hilding A., Brismar K., Hamsten A., Efendic S., Ostenson C. G. (2012). Low serum 25-hydroxyvitamin D level predicts progression to type 2 diabetes in individuals with prediabetes but not with normal glucose tolerance. *Diabetologia*.

[8] Pittas A. G., Dawson-Hughes B., Li T. (2006). Vitamin D and calcium intake in relation to type 2 diabetes in women. *Diabetes Care*.

[9] Pittas A. G., Harris S. S., Stark P. C., Dawson-Hughes B. (2007). The effects of calcium and vitamin D supplementation on blood glucose and markers of inflammation in nondiabetic adults. *Diabetes Care*.

[10] Robinson J. G., Manson J. E., Larson J. (2011). Lack of association between 25(OH)D levels and incident type 2 diabetes in older women. *Diabetes Care*.

[11] Veronese N., Sergi G., De Rui M. (2014). Serum 25-hydroxyvitamin D and incidence of diabetes in elderly people: the PRO.V.A. study. *The Journal of Clinical Endocrinology & Metabolism*.

[12] Yang W., Lu J., Weng J. (2010). Prevalence of diabetes among men and women in China. *The New England Journal of Medicine*.

[13] Zheng T., Gao Y., Baskota A., Chen T., Ran X., Tian H. (2014). Increased plasma DPP4 activity is predictive of prediabetes and type 2 diabetes onset in Chinese over a four-year period: result from the China National Diabetes and Metabolic Disorders Study. *The Journal of Clinical Endocrinology & Metabolism*.

[14] Department of Noncommunicable Disease Surveillance (1999). Definition, diagnosis and classification of diabetes mellitus and its complications: report of a WHO consultation. *Part 1. Diagnosis and Classification of Diabetes Mellitus*.

[15] Jensen C. C., Cnop M., Hull R. L., Fujimoto W. Y., Kahn S. E., American Diabetes Association GENNID Study Group (2002). *β*-cell function is a major contributor to oral glucose tolerance in high-risk relatives of four ethnic groups in the U.S. *Diabetes*.

[16] Scragg R., Sowers M., Bell C., Third National Health and Nutrition Examination Survey (2004). Serum 25-hydroxyvitamin D, diabetes, and ethnicity in the Third National Health and Nutrition Examination Survey. *Diabetes Care*.

[17] Tahrani A. A., Ball A., Shepherd L., Rahim A., Jones A. F., Bates A. (2010). The prevalence of vitamin D abnormalities in south Asians with type 2 diabetes mellitus in the UK. *International Journal of Clinical Practice*.

[18] Chiu K. C., Chu A., Go V. L., Saad M. F. (2004). Hypovitaminosis D is associated with insulin resistance and *β* cell dysfunction. *The American Journal of Clinical Nutrition*.

[19] Lim S., Kim M. J., Choi S. H. (2013). Association of vitamin D deficiency with incidence of type 2 diabetes in high-risk Asian subjects. *The American Journal of Clinical Nutrition*.

[20] Forouhi N. G., Luan J., Cooper A., Boucher B. J., Wareham N. J. (2008). Baseline serum 25-hydroxy vitamin d is predictive of future glycemic status and insulin resistance: the Medical Research Council Ely Prospective Study 1990-2000. *Diabetes*.

[21] Gagnon C., Lu Z. X., Magliano D. J. (2011). Serum 25-hydroxyvitamin D, calcium intake, and risk of type 2 diabetes after 5 years: results from a national, population-based prospective study (the Australian Diabetes, Obesity and Lifestyle study). *Diabetes Care*.

[22] Takiishi T., Gysemans C., Bouillon R., Mathieu C. (2010). Vitamin D and diabetes. *Endocrinology and Metabolism Clinics of North America*.

[23] Berridge M. J. (2017). Vitamin D deficiency and diabetes. *The Biochemical Journal*.

[24] Zemel M. B. (1998). Nutritional and endocine modulation of intracellular calcium: implications in obesity, insulin resistance and hypertension. *Molecular and Cellular Biochemistry*.

[25] Song Y., Wang L., Pittas A. G. (2013). Blood 25-hydroxy vitamin D levels and incident type 2 diabetes: a meta-analysis of prospective studies. *Diabetes Care*.

[26] Ye Z., Sharp S. J., Burgess S. (2015). Association between circulating 25-hydroxyvitamin D and incident type 2 diabetes: a Mendelian randomisation study. *The Lancet Diabetes & Endocrinology*.

[27] Mosekilde L. (2005). Vitamin D and the elderly. *Clinical Endocrinology*.

[28] Mithal A., Wahl D. A., Bonjour J. P. (2009). Global vitamin D status and determinants of hypovitaminosis D. *Osteoporosis International*.

[29] Sinotte M., Diorio C., Bérubé S., Pollak M., Brisson J. (2009). Genetic polymorphisms of the vitamin D binding protein and plasma concentrations of 25-hydroxyvitamin D in premenopausal women. *The American Journal of Clinical Nutrition*.

[30] Hirai M., Suzuki S., Hinokio Y. (2000). Variations in vitamin D-binding protein (group-specific component protein) are associated with fasting plasma insulin levels in Japanese with normal glucose tolerance. *The Journal of Clinical Endocrinology & Metabolism*.

[31] Ortlepp J. R., Metrikat J., Albrecht M., von Korff A., Hanrath P., Hoffmann R. (2003). The vitamin D receptor gene variant and physical activity predicts fasting glucose levels in healthy young men. *Diabetic Medicine*.

[32] He R., Hu Y., Zeng H. (2017). Vitamin D deficiency increases the risk of peripheral neuropathy in Chinese patients with type 2 diabetes. *Diabetes/Metabolism Research and Reviews*.

[33] Damasiewicz M. J., Magliano D. J., Daly R. M. (2013). Serum 25-hydroxyvitamin D deficiency and the 5-year incidence of CKD. *American Journal of Kidney Diseases*.

[34] Luo B. A., Gao F., Qin L. L. (2017). The association between vitamin D deficiency and diabetic retinopathy in type 2 diabetes: a meta-analysis of observational studies. *Nutrients*.

